# Effect of Lead (Pb) on Inflammatory Processes in the Brain

**DOI:** 10.3390/ijms17122140

**Published:** 2016-12-19

**Authors:** Karina Chibowska, Irena Baranowska-Bosiacka, Anna Falkowska, Izabela Gutowska, Marta Goschorska, Dariusz Chlubek

**Affiliations:** 1 Department of Biochemistry and Medical Chemistry, Pomeranian Medical University, Powstańców Wlkp. 72, 70-111 Szczecin, Poland; zara1988@wp.pl (K.C.); anula05@gmail.com (A.F.); rcmarta@wp.pl (M.G.); dchlubek@pum.edu.pl (D.C.); 2 Department of Biochemistry and Human Nutrition, Pomeranian Medical University, Broniewskiego 24, 71-460 Szczecin, Poland; izagut@poczta.onet.pl

**Keywords:** brain, lead (Pb), inflammatory processes

## Abstract

That the nervous system is the main target of lead (Pb) has long been considered an established fact until recent evidence has linked the Pb effect on the immune system to the toxic effects of Pb on the nervous system. In this paper, we present recent literature reports on the effect of Pb on the inflammatory processes in the brain, particularly the expression of selected cytokines in the brain (interleukin 6, TGF-β1, interleukin 16, interleukin 18, and interleukin 10); expression and activity of enzymes participating in the inflammatory processes, such as cyclooxygenase 2, caspase 1, nitrogen oxide synthase (NOS 2) and proteases (carboxypeptidases, metalloproteinases and chymotrypsin); and the expression of purine receptors P2X4 and P2X7. A significant role in the development of inflammatory processes in the brain is also played by microglia (residual macrophages in the brain and the spinal cord), which act as the first line of defense in the central nervous system, and astrocytes—Whose most important function is to maintain homeostasis for the proper functioning of neurons. In this paper, we also present evidence that exposure to Pb may result in micro and astrogliosis by triggering TLR4-MyD88-NF-κB signaling cascade and the production of pro-inflammatory cytokines.

## 1. Introduction

Over the last decade, it has become apparent that the immune system and the nervous system are tied to each other [1,2]. Neural factors regulate the immune system and the balance between cytokines, and immune factors such as cytokines, the major histocompatibility complex neuronal differentiation, and the growth and stabilization of neuronal connections [3]. In the past few years, a number of studies have provided evidence of the important role of inflammatory processes in the pathogenesis of many neurodegenerative disorders such as Alzheimer’s disease, Parkinson’s disease and multiple sclerosis [4,5,6,7]. Exposure to lead (Pb) at different stages of embryonic development has been shown to affect the immune system [8].

Environmental exposure to lead remains a major problem in many countries, and the risk of complications is particularly high in populations living in polluted urban and industrial areas [9,10]. The main target of Pb is the nervous system, with the developing brain appearing to be particularly sensitive to its toxic effects. Results from experiments performed on rodents have indicated that Pb neurotoxicity could pose significant health problems in children [11,12,13]. Pb easily penetrates into the developing neural tissue [14], directly affecting the neurons and synapses, or indirectly affecting neuronal connections via glial cells [15]. Pb neurotoxicity may manifest in children as a decrease in IQ, or disorders of behavior, memory or learning [16,17]. The mechanisms underlying the neurotoxic action of Pb are those related, for example, to cytotoxicity, changes in the storage and release of neurotransmitters, energy metabolism disorders, induction of apoptosis, inflammation and oxidative stress [18]. Although the history of research on Pb neurotoxicity is relatively long, the pro-inflammatory effect of Pb in the brain has not been fully explored, despite the fact that this effect has been reported in many other cells, tissues and organs [19]. Studies in humans, animals and in cell cultures describe Pb-induced increases in the concentrations of inflammatory mediators [20,21].

## 2. Effects of Lead on the Expression of Cytokines in Brain

Under physiological conditions, cytokine expression in the central nervous system is very low or undetectable, but tends to increase after the occurrence of pathological stimuli, such as trauma, infections, autoimmune diseases or exposure to toxic agents [4,22]. Cytokines released by glial cells contribute to initiation of processes that lead to the death of neurons [23,24].

### 2.1. Expression of Interleukin 6 and TGF-β1

Kasten-Jolly et al. studied the effect of 0.1 mM lead acetate (PbAc) on the gene expression of some cytokines in the central nervous system. Mouse pups were exposed to Pb from the 8th day of pregnancy to 21 days after birth, i.e., the period when the pups’ mothers were given drinking water with 0.1 mM PbAc [15]. That exposure to 0.1 mM PbAc resulted in Pb levels in the pups’ blood of 15–20 µg/dL [15]. Among the cytokines examined, a significant PbAc-induced change was observed in the gene expression of *interleukin 6* (*IL-6*) and *transforming growth factor β1* (*TGF-β1*) [15], with the gene expression of *IL-6* higher in each region of the brain, i.e., in the frontal cortex, cerebellum, hypothalamus, striatum, hippocampus and substantia nigra, in comparison with the untreated control [15]. Significantly, the overexpression of IL-6 in the development of the brain could adversely affect the growth and differentiation of neurons via reactive gliosis (increased size and the number of astrocytes and ramified microglia), and may have an activating effect on *N*-methyl-d-aspartate receptors (NMDA) receptors in neurons, causing excessive activation of nerve cells, which in turn would lead to their death by necrosis [15,25,26]. At the same time, exposure to 0.1 mM PbAc had no effect on IL-6 protein in the frontal cortex, cerebellum, and hypothalamus, and reduced levels of IL-6 were observed in the striatum, hippocampus and substantia nigra [15]. However, as indicated by those authors, the discrepancy between the levels of mRNA and IL-6 protein levels in the various brain areas was probably caused by the method of protein detection, which measured only the free form of IL-6 and not IL-6 bound with its receptor [15].

In the same research, the gene expression of *TGF-β1* in the group exposed to 0.1 mM PbAc was greatest in the frontal cortex [15]. The levels of TGF-β1 protein were elevated in the frontal cortex and cerebellum, and reduced in the substantia nigra [15]. Slightly reduced levels of TGF-β1 were also observed in the striatum, hippocampus, and hypothalamus, but those changes were not significantly different from the control group [15]. In a study by Wyss-Coray et al., the over-production of TGF-β1 by astroglial cells resulted in the stimulation of inflammatory processes in the central nervous system of transgenic mice [27], which, combined with the results of a study conducted by Kasten-Jolly et al. confirm the role of Pb in inflammatory processes in the central nervous system through the above-described effect on the gene expression of *TGF-β1*.

#### 2.1.1. The Mechanism of the Effect of Pb on the Gene Expression of *IL-6* and *TGF-β1*

As mentioned above, Kasten-Jolly et al. demonstrated that 0.1 mM PbAc had a significant effect on gene expression of the cytokines *IL-6* and *TGF-β1* [15]. The likely molecular mechanism of the effect of PbAc on the gene expression of cytokines *IL-6* and *TGF-β1* starts with Pb penetrating the cell, mobilization of calcium ions, cleavage of phosphatidylinositol bisphosphate (PIP2) into inositol trisphosphate (IP3) and diacylglycerol (DAG), activation and migration of PKC to the cytoplasmic membrane, and consequent transcription of *c-jun* and *c-fos* genes. Kasten-Jolly et al. confirmed that 0.1 mM PbAc increases the gene expression of *c-jun* and *c-fos* (early response genes) and the production of c-jun and c-fos proteins which led to the formation of the nuclear transcription factor AP-1 (activator protein 1) via dimerization [15,28]. Before dimerization, the c-jun and c-fos proteins must be phosphorylated; the mitogen-activated protein kinase (MAPK) pathway plays a role in the signal transduction pathway, and Pb-activated PKC affects the system of MAP kinases [29,30]. It has been shown that exposure to 0.1 mM PbAc significantly increases the gene expression of *MAPK6*, *Mapk11 (p38) Map4k5*, *Map4k6* and *MAPkapk2* [15].

Promoters of *IL-6* and *TGF-β1* genes have at least one binding site for AP-1, recognizing the TGACTCA sequence [31,32]. Furthermore, the promoters of *IL-6* and *TGF-β1* genes seem to contain a site for the transcription factor SP-1 (specificity protein 1) recognition sequence GGGCGG [33,34]. Atkins et al. [35] showed that Pb affects transcription factor SP-1 by interfering with PKC α and MAP kinases.

**Figure 1:**
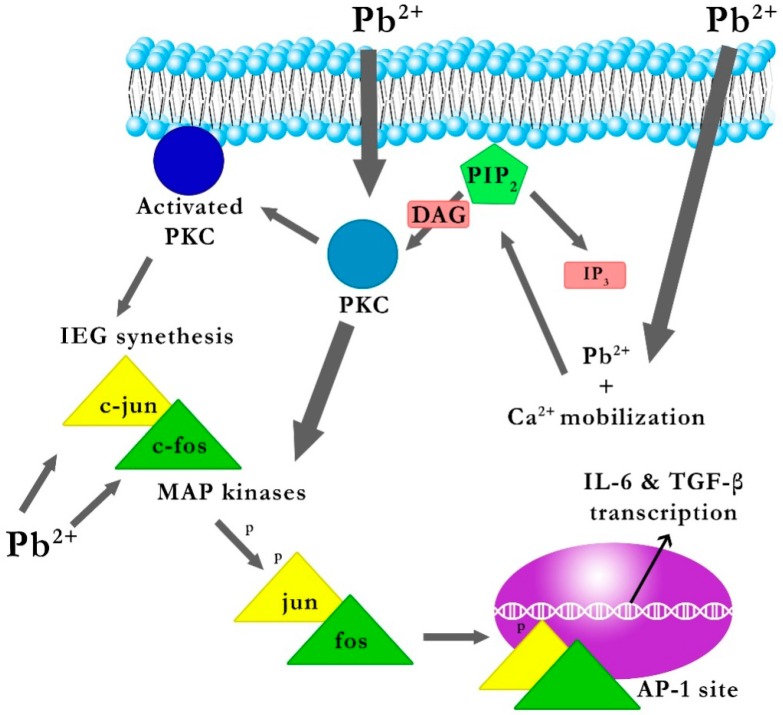
The explanation of the likely effect of Pb on the gene expression of *IL-6* and *TGF-β1* [22]. PIP2: phosphatidylinositol; IP3: 4,5-bisphosphate inositol 1,4,5-trisphosphate; DAG: diacylglycerol; PKC: protein kinase C; IEG: immediate early gene; *c-jun and c-fos* genes; AP-1: activator protein 1; MAP kinases: mitogen-activated protein kinases. Penetrating into the interior of the cell, Pb mobilizes cellular calcium, which leads to the cleavage of phosphatidylinositol 4,5-bisphosphate (PIP2) into inositol 1,4,5-trisphosphate (IP3) and diacylglycerol (DAG), activation and migration of protein kinase C (PKC) to the cytoplasmic membrane, and consequently leads to the transcription of the immediate early gene (IEG) *c-jun* and *c-fos* genes, and production of c-jun and c-fos proteins whose dimerization leads to the formation of the nuclear transcription factor activator protein 1 (AP-1). Prior to dimerization, proteins c-jun and c-fos need to be phosphorylated by mitogen-activated protein kinase (MAPKs), where the Pb-activated PKC influences the MAPK system. Promoters of *IL-6* and *TGF-β1* genes have at least one binding site for AP-1, which in turn results in increased gene expression of *IL-6* and *TGF-β1* genes.

In conclusion, it seems that Pb may increase the transcription of the aforementioned genes, if the genes of *IL-6* and *TGF-β1* had a site for one of the regulatory elements AP-1 or SP-1 [15] (Figure 1).

#### 2.1.2. Effect of Pb on IL-6 and TGF-β1 Signal Transduction Pathways

Synthesized cytokines IL-6 and TGF-β1 are secreted by the cell and bind with appropriate target receptors. By annealing to its receptor, IL-6 Rα (a protein complex consisting of a subunit of the IL-6 receptor and gp-130), IL-6 activates Janus kinase (JAK1, JAK2, and TYK2) associated with the membrane gp-130 [36], resulting in tyrosine phosphorylation on gP-130, which subsequently recruits molecules such as SHP2 or STAT3 [37]. When bound to gp-130, STAT3 is phosphorylated by JAK kinase, which results in the dimerization of STAT3 (homo or heterodimer of STAT1) and translocation to the nucleus, where it influences gene expression by binding with appropriate DNA sequences [15]. Kasten-Jolly et al. showed that 0.1 mM PbAc slightly increases the gene expression of STAT3, thus indicating the possible impact of Pb on signal transduction by IL-6 after binding to IL-6 Rα [15].

**Figure 2:**
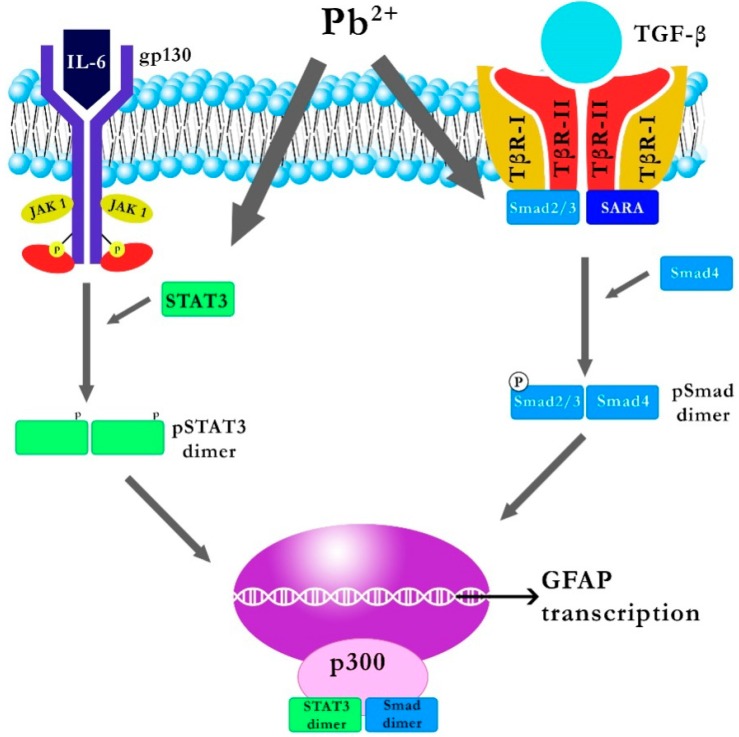
Explanation of the likely impact of Pb on the IL-6 and TGF-β1 signal transduction pathways [22]. STAT3: signal transducer and activator of transcription; JAK1: Janus kinase 1, GFAP: glial fibrillary acidic protein, Smad: receptor-regulated protein. By annealing/binding to its receptor IL-6 Rα, IL-6 activates JAK associated with the membrane gp-130, which results in the phosphorylation of tyrosine on gp-130 which then recruits molecules such as signal transducer and activator of transcription 3 (STAT3). When connected to gp-130, STAT3 is phosphorylate by Janus kinase (JAK kinase), which results in the dimerization of STAT3 and translocation to the nucleus, where, by combining with the appropriate DNA sequences, it influences the expression of multiple genes. Binding of TGF-β to TβRII activates its kinase subunit, increases affinity to TβRI, and consequently the activation of TβRI which stimulates the phosphorylation of receptor-regulated protein Smad-3 bound to 3-endosomal SARA. The phosphorylated Smad-3 forms a heterodimer with Smad-4, and the resulting complex is transported to the nucleus, where it combines with CAGAC sequences or sequences rich in GC pairs in the promoter regions of many genes.

Connection of TGF-β to TβRII (type II TGFβ receptor) activates this kinase subunit, increases affinity to TβRI (type I TGFβ receptor) and, consequently, activates TβRI which stimulates phosphorylation of the Smad-3 protein attached to the 3-endosomal SARA protein [38]. The phosphorylated Smad-3 protein forms a heterodimer with Smad-4, and the resulting complex is transported into the nucleus, where—via the MH1 domain—it binds to CAGAC sequences or sequences rich in GC pairs in the promoter regions of many genes [38,39]. One of them is the *GFAP* gene encoding the GFAP astrocyte protein whose production is controlled by factors such as STAT3 and Smad; interestingly, binding to STAT3 or Smad separately does not induce transcription of the *GFAP* gene; transcription is possible only if the Smad and STAT3 are both associated with the *GFAP* gene, and a P300 molecule serves as the link between them [40]. Increasing the transcription of the *GFAP* gene and other genes can cause an increase in the number of astrocytes, which can interfere with the balance of development between astrocytes and neurons, and may interfere with the system of neuronal connections [15]. Kasten-Jolly et al. [15] showed that 0.1 mM PbAc results in increased gene expression of *Smad-3*, the product of which (as mentioned above) is a substrate for TβRI, thus indicating the possible effect of Pb on the transduction of TGF-β when attached to TβRII [38] (Figure 2).

### 2.2. Effect of Pb on Interleukin 16 Expression

Some cytokines have their neuronal forms, for example IL-16’s elongated form nIL-16, acting selectively on neuronal ion channels (its presence has been detected only in neurons of the hippocampus and cerebellum) [41]. A shortened form of IL-16 acts as a chemokine for T CD4+ cells [15]. In an aforementioned study by Kasten-Jolly et al. [15] a slight increase in IL-16 gene expression resulted from exposure of mouse pups to 0.1 mM PbAc from Day 8 of pregnancy up to 21 days after birth.

### 2.3. Effect of Pb on Interleukin 18 Expression

IL-18 is a pro-inflammatory cytokine that, in glial cells isolated from mice, induces intracellular gene expression of *IL-1α* and *IL-1β* and the release of IL-6, indicating that IL-18 may contribute to the development of inflammation in the brain [42].

Previous studies on the gene expression of *IL-18* have suggested that this cytokine is constitutively expressed in astrocytes and microglial cells [43]. However, the study by Kasten-Jolly et al. [15] showed that IL-18 mRNA and IL-18 protein levels increase in the frontal cortex of the brain of mouse pups following exposure to 0.1 mM PbAc from the 8th day of pregnancy to the 21st day after birth [15]. An increase in *IL-18* gene expression in the frontal lobe of the brain was noted in certain neurodegenerative disorders [44]. An in vitro study by Curran et al. [45] also demonstrated that IL-18 weakens the long-term potentiation (LTP) through the NMDA receptor, most likely through activation of the p38 MAPK cascade that includes Map4k6 and Mapkapk2 [45]. All of these kinases are up-regulated following exposure to Pb, thus indicating a potential molecular mechanism of Pb effect on the above-mentioned processes [15,45].

Research conducted by Cordova et al. [46] also showed that Pb can stimulate phosphorylation of p38 MAPK cascade in the hippocampus of immature rats.

### 2.4. Effect of Pb on Interleukin 10 Expression

Wong et al. [47] showed the anti-inflammatory effect of IL-10 through its ability to directly block gene expression of *IL-1*. Kasten-Jolly et al. [3] examined the level of anti-inflammatory cytokine IL-10 in selected regions of the rat brain (i.e., cortex, cerebellum, hypothalamus, striatum, hippocampus and substantia nigra) following exposure to 0.1 mM PbAc. The results showed a statistically significant decrease in interleukin 10 in the area of the cerebral cortex. Thus, a decrease in the concentration of IL-10 in the cerebral cortex upon exposure to 0.1 mM PbAc is consistent with the supposition that Pb promotes the development of the inflammatory response in this region of the brain [3].

## 3. Effects of Pb on Enzymes

### 3.1. Effects of Pb on the Gene Expression and Activity of Cyclooxygenase-2 (COX 2)

COX-2 catalyzes the conversion of free arachidonic acid (AA) to prostaglandin H2 (PGH2), which is then converted in a series of enzymatic and non-enzymatic mechanisms to the primary prostanoids, PGE2, PGF2α, PGD2, PGI2, and TXA2, and simultaneously generated ROS [48]. In the peripheral tissues, COX-1 is a constitutive form of cyclooxygenase, while COX-2 is an inducible form [49]. In contrast, COX-2 is the basic form expressed in neurons [50,51,52]. Immunoreactivity of COX-2 has been identified under the forebrain, including the dentate gyrus granule cells, pyramidal cells in the hippocampus, piriform cortex, the superficial cell layers of neocortex, and amygdala cells [53]. In pathological conditions, such as hypoxia/ischemia and seizures, as well as neurodegenerative diseases including Alzheimer’s disease (AD), it has been demonstrated that over expression of COX-2 is associated with neurotoxicity [54]. Wei et al. [49] investigated the effect of various doses of Pb (from 25 to 100 μM) on the induction of COX-2 in rat C6 glioma cells, mouse BV2 microglia, in primary cultures of cortex neurons, in neural stem cells (NSCs) and RBE4 cells (brain endothelium). The results of that research showed that Pb causes an induction of COX-2 in C6, BV2, primary culture of cortical neurons and NSCs [45]. In RBE4 cells, Pb (at doses greater than 50 μM) caused only a slight increase in *COX-2* gene expression [49].

Previous studies have shown that in exposure to heavy metals such as mercury and arsenic, the *COX-2* gene is regulated by transcription factors such as NF-κB, AP-1, and NFAT [55,56,57,58,59]. Wei et al. [49] investigated the influence of Pb on the induction of *COX-2* gene transcription in a mechanism mediated by transcription factors NF-κB, AP-1 and NFAT. In that study, only the NFAT transcription factor was up-regulated by exposure to Pb [49]. In order to confirm the critical role of transcription factor NFAT in the induction of gene transcription of *COX-2* by Pb, the authors mutated NFAT binding sites within the promoter of the *COX-2* gene, which resulted in the abolition of *COX-2* gene transcription, thus confirming that NFAT plays a key role in the transcription of the *COX-2* gene induced by Pb in glial cells. That observation may give rise to the use of molecular inhibitors of COX-2 in attempts to remove the neurotoxic properties of Pb [49].

### 3.2. Effect of Pb on the Gene Expression and Activity of Caspase-1 and NOS 2

Caspase-1 (enzyme converting IL-1β, ICE) is the enzyme which converts the precursors of cytokines such as IL-1β and IL-18 to the mature active forms which play a key role in inflammatory processes that increase with aging [60]. By controlling the production of the bioactive form of IL-1β, caspase-1 causes an up-regulation of the gene expression of *nitric oxide synthase* (*iNOS or NOS2*) and thus increases the production of nitric oxide (NO) [61]. Then the released NO (primarily by activated glial cells) causes a change in proteins, DNA, RNA, lipids, impairing cellular function and consequently leading to cell death around the activated glial cells [3]. Kasten-Jolly et al. [3] investigated the influence of Pb on the gene expression of *caspase-1* and *NOS2* in the central nervous system [3]. The survey observed the up-regulation of the gene expression of *caspase-1* and *NOS2* upon exposure to 0.1 mM PbAc during brain development [3]. Ramesh et al. [62] also observed increased NOS2 activity in rats exposed to subchronic doses of Pb [62].

Significantly, in the study by Sifringer et al. [63] the over expression of caspase-1 in cerebral ischemia had a destructive effect on brain tissue, and the blockage of this enzymes’ expression reduced the area of cerebral infarction.

### 3.3. Effects of Pb on the Expression of Proteases

The symptoms of ongoing inflammation include increased protease expression [64]. The results of a study conducted by Kasten-Jolly et al. [3] showed that exposure to 0.1 mM PbAc resulted in increased gene expression of many catabolic enzymes, such as carboxypeptidases, metalloproteinases and chymotrypsin.

## 4. Effects of Pb on Microglia and Astroglia

Microglia are glial cells that are residual macrophages in the brain and spinal cord and act as a first line of defense in the central nervous system [65]. A study by Liu et al. [66] on the effect of Pb on the activation of microglia and on the inflammatory response in the hippocampus of young mice, examined the expression of Iba1 (ionized calcium binding adapter molecule 1) and TLR4 (Toll-like receptor 4), both of which are markers of activated microglia, MyD88 (myeloid differentiation factor 88), NFκB (nuclear factor-kappa B), IL-1β (interleukin-1 beta) and TNFα (tumor necrosis factor α) [66]. Young mice were divided into four groups: a control group, a group with inhibitory peptide to MyD88, a Pb exposed group, and a group with an inhibitory peptide to MyD88 and Pb [66]. Mice in the Pb group were given intraperitoneally PbAc 15 mg/kg for three days, and inhibitory peptide to MyD88 administered separately (group with the inhibitory peptide to MyD88) or by injection into the cerebral ventricle (group with inhibitory peptide to MyD88 and Pb) and the control group was injected saline [66]. Exposure to Pb caused a significant increase in the number of immunoreactive microglial cells (Iba1+) and an increase in the number of immunoreactive microglial cells (Iba1+/TLR4+) dispersed/scattered in the dentate gyrus of the hippocampus compared to control [66]. It is known that microglia can initiate an innate immune response via TLRs located on their surface. Rolls et al. demonstrated that a type of TLR located on NPCs-neural stem/progenitor cells influences neurogenesis in the hippocampus; a deficiency of TLR2 in the mice impairs neurogenesis in the hippocampus, and the absence of TLR4 results in increased proliferation and differentiation of cells [67,68]. Activation of TLRs results in increased synthesis of pro-inflammatory cytokines (IL-1, -6, -8, and -12) and TNF-alpha [69]. Furthermore, the stimulation of a TLR (TLR4) increases the phagocytic capacity of macrophages and increases the synthesis of nitric oxide (NO) and reactive oxygen species [69]. Signal transduction by TLRs involves proteins such as MyD88, IRAKs (IL-1-1R1-associated protein kinases), TAK1 (TGF-β-activated kinase), TABs (TAK1-binding proteins) and TRAF6 (TNF-receptor associated factor 6) [70].

Upon TLR stimulation, the MyD88 component connects directly to the cytoplasmic TIR domain of TLR (this refers to TLR5, TLR7, and TLR9) or via the TIRAP adapter protein (TLR2 and TLR4). A study by Liu et al. [66] on a group of mice exposed to PbAc observed elevated expression of TLR4 and MyD88 compared to the control group. This is followed by the activation of IRAK-4, which results in the phosphorylation of IRAK-1. Active IRAK-1 binds to TRAF6, thereby allowing the activation of the TAK1/TAB complex. The activated complex TAK1/TAB activates IκB kinase (IKK) and MAP kinase [71,72,73,74,75].

p38 MAPK and ERK1/2 (belonging to the MAPK family) work actively in microglia, astrocytes and neuronal cells, and mediate the activation of transcription factors CREB and AP-1, which—in turn—involve target genes [66,76]. In a study by Liu et al. [66] the total expression of p38 MAPK and ERK1/2 was significantly increased in the group exposed to the PbAc, indicating that activation of p38 MAPK and ERK1/2 signaling pathways may be involved in TLR signal transduction in the hippocampus of young mice exposed to Pb [66].

In turn, activated IKK leads to phosphorylation and degradation of IκB in the proteasome, and thereby the release of NF-κB complex from NF-IKB-κB. The above-mentioned processes enable the translocation of NF-κB to the nucleus, where the expression of genes encoding inflammatory cytokines is induced [77].

**Figure 3:**
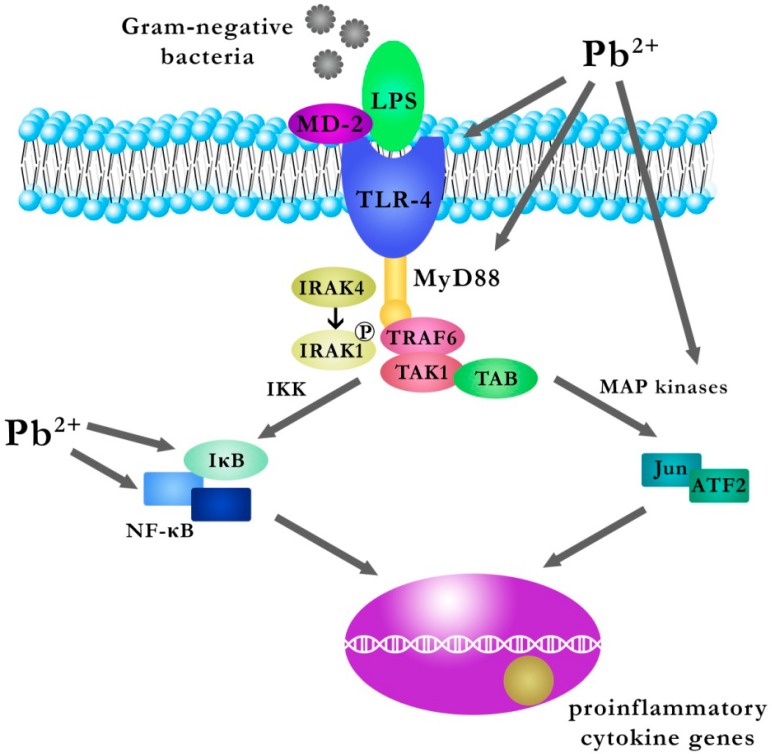
The effect of Pb on the signaling pathway TLR4-MyD88-NF-κB and the production of pro-inflammatory cytokines in microglial cells. LPS: lipopolysaccaride; MD-2: myeloid differentiation 2; TLR-4: toll like receptor 4; MyD88: myeloid differentiation primary response gene 88; IRAK: IL-1R-associated kinase; TRAF6: TNF receptor-associated factor-6; TAB: TAK1 binding proteins; ATF2: activating transcription factor; IKK: IκB kinase, TAK1: kinase transforming growth factor-β-activated kinase 1; MAP kinases: mitogen-activated protein kinases [66]. Microglial cells can initiate an innate immune response by TLRs located on their surface. Upon the stimulation of toll like receptor 4 (TLR-4), myeloid differentiation primary response gene 88 (MyD88) binds to the cytoplasmatic domain of TLR-4. This is followed by the activation of IL-1R-associated kinase (IRAK-4), which results in the phosphorylation of IRAK-1. Activated IRAK-1 binds to TNF receptor-associated factor-6 (TRAF6 factor), thus allowing the activation of the TAK1/TAB complex kinase transforming growth factor-β-activated kinase 1/ TAK1 binding proteins. The activated TAK1/TAB complex activates IκB kinase (IKK) and mitogen-activated protein kinases (MAP kinases). The activated IKK kinase leads to the phosphorylation and degradation of IκB in the proteasome, and thereby the release of NF-κB from the NF-IKB-κB complex. The above-mentioned process enables the translocation of NF-κB to the nucleus, where the expression of genes encoding pro-inflammatory cytokines is induced.

A study by Liu et al. [66] reports a significantly increase in the number of microglia NFκB in the group exposed to PbAc compared with the control group. Western blot analysis also confirmed a significant increase in the expression of p-IκB and NF-κB in the group exposed to PbAc compared with the control group, thereby indicating that Pb exposure activated NFκB signaling in the mouse hippocampus [66]. In that study on a group of mice exposed to PbAc, there was also a significant increase in the expression of IL-1β and TNF-α in the hippocampus (the authors indicate that activated microglial cells could be a major source of these cytokines), confirming a Pb-induced increase in the production of pro-inflammatory cytokines [66]. Furthermore, the results of that study also suggest that exposure to Pb may cause not only microgliosis but also astrogliogenesis, as neural precursor cells differentiated into astroglia instead of nerve cells in the hippocampus of the young Pb-exposed mice via triggering the TLR4-MyD88-NF-κB signaling cascade and the production of pro-inflammatory cytokines. This is very significant in the long-term consequences of neurogenesis and functional plasticity in the brain, in which astroglial and microglial cells play an important regulatory role [66,78]. The authors indicate that administration of inhibitory peptide MyD88 may substantially mitigate the neurotoxic effects caused by Pb [66] (Figure 3).

It is known that the most important function of astroglia cell is maintaining homeostasis for the proper functioning of neurons [19]. Some neurotoxic agents, including Pb, induce an astrocyte response in which glial cells undergo rapid changes [19,79,80]. The response of astrocytes, i.e., reactive gliosis is associated with morphologic changes inside the cell and increased synthesis of many proteins [81,82]. One of the most important features related to this phenomenon is an increase in the expression of two glial markers, GFAP and S-100β [19]. GFAP is not specific to astrocytes, but also to oligodendrocytes [83]. At physiological concentrations, S-100β acts as a neurotrophic factor during brain development [84], and in more extreme pathological concentrations it can act as a pro-inflammatory cytokine and can contribute to the development of neuroinflammatory response resulting in neuronal dysfunction, manifested as a reduced expression of axonal markers (synapsin I and synaptophysin) [85].

A study by Strużyńska et al. [19] showed increased production of pro-inflammatory cytokines and axonal damage, with concurrent astrocytic activation following exposure of the immature rat brain to PbAc. For this purpose, rat pups of either sex from 15 days of age were intraperitoneally injected with PbAc daily at a dose of 15 mg/kg (group Pb) or saline (control group) for two weeks [19]. The administration schedule used in that experiment resulted in an increase in blood Pb levels close to those typical for long term exposure to Pb (3.3 µg/dL in the control group and 30.8 µg/dL in the Pb group) [19]. That research observed increased expression of GFAP and S-100β as a result of exposure to Pb in the hippocampus, cerebellum and forebrain cortex, with the largest increase for the S-100β protein in the hippocampus [19].

One of the most potent inducers of reactive astrogliosis is IL-1β, produced by activated microglial cells and astrocytes [86,87]. IL-1β is a specific cytokine involved in the communication between glial cells during brain damage, and plays a key role in the regulation of inflammatory processes [19]. Strużyńska et al. [19] also examined Pb-exposed rats in terms of the expression profile of pro-inflammatory cytokines in the brain. In the forebrain and hippocampus, but not in the cerebellum, they detected increased expression of pro-inflammatory cytokines, but the cytokine secretion profiles were different. In the hippocampus it showed an increase in IL-1β and TNF-α, while in the forebrain cortex showed an increase in IL-6 [19]. Increased IL-1β after administration of Pb signals the induction of mechanisms leading to the inflammatory cascade and points to the potential pro-inflammatory effect of Pb [19].

Fractalkine (CX3CL1) is a chemokine that exhibits high expression in neurons and astrocytes, and its receptor (CX3CR1) is present in neurons and microglial cells [19]. It is released from astrocytes and microglial cells stimulated by TNF-alpha and interferon-gamma [88]. In a study by Chapman et al. [89] fractalkine released from cultured neurons intensified microglia chemotaxis [89]. Another function of CX3CL1 is a neuroprotective role, which consists in the inhibition of neuronal cell death by reducing the production of: (i) TNF-alpha and other inflammatory cytokines; and (ii) NO by activated microglial cells [90,91]. The level of fractalkine expression in the study by Strużyńska et al. [19] was elevated in all brain regions, especially in the forebrain and hippocampus (the weakest immunoreactivity was observed in the cerebellum) [19]. The presence of fractalkine in the hippocampus and forebrain as a result of exposure to Pb could indicate either its neuroprotective role in these regions, or its pro-inflammatory nature acting as a chemo-attractant for macrophages [19]. In light of these data, the role of fraktalkine released in the brains of rats exposed Pb needs further study.

**Figure 4:**
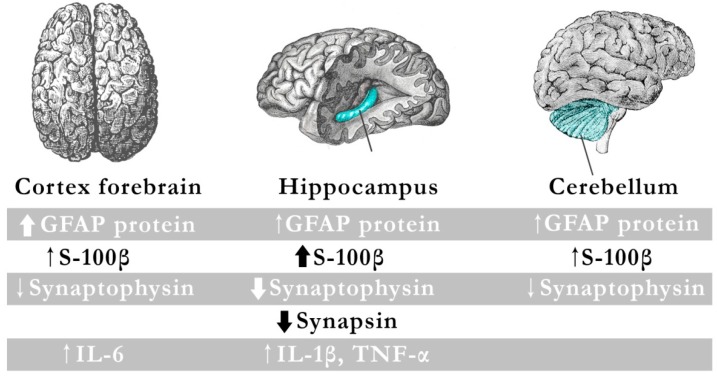
Inflammation-like glial responses in Pb-exposed immature rat brain. The diagram shows the results of a study conducted by Strużyńska et al., in which immature rat brains were exposed to lead. The objective was to demonstrate the presence of increased production of pro-inflammatory cytokines and axonal damage, with a concurrent astrocytic activation following exposure to lead. In all three studied brain regions (cerebellum, hippocampus, and cortex forebrain), they observed increased expression of GFAP and S-100β, with the most significant reduction in the expression of axonal markers (synapsin I and synaptophysin) observed in the hippocampus. In addition, IL-1β and TNF-α increased in the hippocampus, while IL-6 increased in the forebrain cortex.

In summary, exposure to Pb during postnatal maturation results in the activation of glial cells with concomitant inflammation and neurodegeneration. This was most pronounced in the hippocampus of the brain of the immature rats. Other studies also confirm the Pb-induced dysfunction of neurons and glial cells in the early stages of development [92]. As increasing evidence shows that neurological inflammation contributes to the pathogenesis and progression of many disorders, it may be assumed that Pb can affect immunological processes in the brain (Figure 4) [19].

## 5. Effects of Pb on the Expression of P2X4 and P2X7

Purine receptors are a large family of receptors of which P2X receptors are a class of membrane ion channels which open in response to binding of extracellular ATP (an agonist) [11]. It has been shown that P2X receptors have an impact on synaptic transmission [93], and in pathological conditions extended activation of purine receptors by high levels of ATP leading to the release of glutamate and ATP [94,95], lead to the development of inflammatory reactions [96] and cell death by necrosis or apoptosis [97]. P2X receptors have seven isoforms, i.e., P2X1–P2X7 [98]. In the central nervous system, P2X7 receptors are found on microglial cells, Schwann cells, as well as on astrocytes [99]. In the peripheral sensory ganglia of rats, P2X7 receptors seem to be located on the glial cells [100].

P2X7 receptor is a mediator in acute brain injury, as the synthesis and location of P2X7 membrane receptor rapidly increases in response to various stimuli. Activation of P2X7 receptors results in damage-associated molecular pattern (DAMP) initiating neuroinflammatory cascades. Further, the formation of the P2X7 receptor pore appears to be necessary for activating the inflammasome. By engaging in a variety of signaling pathways, P2X7 receptors trigger a rapid release of pro-inflammatory cytokines, including TNF-α and IL-1 β and a rapid activation of caspase-1 [101].

Activation or over expression of the P2X7 receptor has also been implicated in damage to motor neurons [102], and blockage of the P2X7 receptor by ATP has shown a neuroprotective effect in animal models of multiple sclerosis [103], Huntington’s disease [104] and in Alzheimer’s disease [105]. Ning et al. [11] examined the expression of P2X7 receptor and synaptophysin in the hippocampus of young mice exposed to Pb [11]. The mice were randomly divided into four groups, with the control group given distilled water, while the remaining three groups given PbAc at concentrations of 0.1%, 0.5% and 1% [11]. Exposure through drinking water containing PbAc began from the beginning of pregnancy and continued until weaning, i.e., 21st day after birth [11]. There was a significant increase in Pb in the blood and hippocampus in the groups exposed to PbAc compared with the control group [11]. P2X7 receptor expression was significantly higher in the groups that received PbAc [11].

Baranowska-Bosiacka et al. [12] also observed an increased expression of P2X7 receptor in a fraction of glia in immature rat brains exposed to Pb [12]. In another study, Baranowska-Bosiacka et al. [65] showed the effect of prenatal and perinatal exposure to Pb on the expression of mRNA and P2X4 and P2X7 proteins, and on the activity of astrocytes (GFAP) and microglia (Iba1) in selected structures of the mesolimbic system (striatum, hippocampus, prefrontal cortex) in rats with tolerance to the antinociceptive effect of morphine [65]. In that study pregnant female rats in the experimental group received 0.1% PbAc in drinking water, starting on the first day of pregnancy until weaning, while females of the control group received tap water [65]. Offspring were weaned at 21 days of age and placed in separate cages. From 21 until 28 days of age the young rats exposed to Pb continued to receive 0.1% PbAc. From 29 to 60 days of age the young rats from both groups received only distilled water. The experiment on the effect of Pb on morphine tolerance in this study was performed on the 60-day-old rats [65]. For that purpose, the animals were divided into: a control receiving 0.9% NaCl; a second group of rats which had already received PbAc during the pre- and neonatal period; a third group of rats receiving morphine (10 mg/kg intraperitoneally); and a fourth group of rats receiving PbAc and morphine (10 mg/kg i.p.) [65]. The exposure to Pb in that study caused an increase in whole blood Pb (20.50 µg/dL) in the group exposed to Pb in comparison with the control group [65]. Pre and neonatal exposure to Pb resulted in up-regulation of the expression of P2X4 receptor in the striatum and prefrontal cortex and P2X7 receptor in the striatum and hippocampus [65]. It was also shown that the expression of GFAP in the striatum and prefrontal cortex and the expression of Iba1 in the striatum and hippocampus were significantly enhanced, indicating the activation of glial cells in pre- and neonatal exposure to Pb [65].

## 6. Conclusions

Although recent advancements in research have resulted in new insights into the molecular mechanisms of Pb toxicity, relatively few papers have concerned the effects of Pb compounds on inflammatory processes in the brain. The existing reports suggest that the presence of Pb in the brain causes a potential pro-inflammatory effect on the central nervous system and neuronal death may be related to the production of various cytokines and chemokines [19]. As described above, the effect of Pb on the inflammatory processes in the brain is omnidirectional and not yet fully understood. Pb affects, among others, the expression of cytokines; the expression and activity of enzymes involved in inflammation such as cyclooxygenase 2, caspase-1, nitric oxide synthase (NOS 2), proteases; and influences the expression of purine receptors P2X4 and P2X7. The effect of Pb on astroglial cells and microglial cells also plays an important role in the development of inflammatory processes.

Undoubtedly, this subject requires further research due to consistent environmental pollution with heavy metals and the harmful effects of even small doses of Pb. It is presently believed that there is no threshold level for Pb toxicity; even very low blood Pb may have an adverse effect, especially on young organisms [106].
